# The Effectiveness and Adverse Events of Amitriptyline and Aripiprazole in Very Elderly Patients With BMS

**DOI:** 10.3389/fpain.2022.809207

**Published:** 2022-03-04

**Authors:** Motoko Watanabe, Chihiro Takao, Zhenyan Liu, Gayatri Nayanar, Takayuki Suga, Chaoli Hong, Trang Thi Huyen Tu, Tatsuya Yoshikawa, Miho Takenoshita, Haruhiko Motomura, Takahiko Nagamine, Akira Toyofuku

**Affiliations:** ^1^Department of Psychosomatic Dentistry, Graduate School of Medical and Dental Sciences, Tokyo Medical and Dental University, Tokyo, Japan; ^2^Department of Basic Dental Sciences, Faculty of Odonto-Stomatology, University of Medicine and Pharmacy at Ho Chi Minh City, Ho Chi Minh City, Vietnam; ^3^Department of Psychiatric Internal Medicine, Sunlight Brain Research Center, Yamaguchi, Japan

**Keywords:** burning mouth syndrome, elderly patients, antidepressants, amitriptyline, aripiprazole, adverse events, pain management, psychopharmacology

## Abstract

Burning mouth syndrome (BMS) is defined by chronic oral burning sensations without any corresponding abnormalities. Besides amitriptyline, aripiprazole has been reported as a possible medication to manage BMS. However, especially for elderly patients, the adverse events of these medications would be a problem. The aim of the present study was to investigate the differences in the effectiveness and adverse events of amitriptyline and aripiprazole in very elderly patients with BMS. This is a retrospective comparative study of 80 years old and older patients with BMS who were initially treated with amitriptyline or aripiprazole and who were new outpatients of our department from April 2017 to March 2020. All clinical data, including sex, age, comorbid physical diseases, comorbid psychiatric disorders, the prescribed doses (initial, maximum, and effective dose), prognosis, and adverse events, were collected from their medical charts. Each medication was selected considering their medical history. Amitriptyline was prescribed in 13 patients (11 women, 82.3 ± 2.1 years old) and aripiprazole was prescribed in 27 patients (26 women, 84.2 ± 3.8 years old). There were no significant between-group differences in sex, age, duration of illness, pain intensity, salivation, and psychiatric comorbidity at the first examination. Amitriptyline clinically improved more patients (7 patients, 53.8%) with the effective dose of 10 (7.5, 15.0) mg than aripiprazole (11 patients, 40.7%) of which the effective dose was 1.0 (0.5, 1.5) mg, although there were no significant between-group differences. The adverse events of amitriptyline were found in 9 patients (69.2%) and most patients had constipation (46.2%). For aripiprazole, 7 patients (25.9%) showed adverse events, most of them reported sleep disorder (11.1%). Amitriptyline had significantly longer duration taking medication (*p* = 0.021) and lower discontinuation (*p* = 0.043) despite of higher occurrence rate of adverse events (*p* = 0.015) compared to aripiprazole. These results suggest that both psychopharmacotherapies with a low dose of amitriptyline and aripiprazole are effective for the very elderly patients with BMS. Furthermore, aripiprazole may have some advantages in the adverse events compared to amitriptyline; however, the low dose amitriptyline monotherapy may have more benefit in the effectiveness and tolerability over prudent collaboration with primary physicians.

## Introduction

Burning mouth syndrome (BMS) is defined by chronic burning sensations in the oral cavity without any corresponding abnormalities (1). For the psychopharmacotherapy of BMS, amitriptyline is a first-line medication (2), and aripiprazole has been reported as another effective medication for the treatment of BMS. These medications also induce adverse events in some patients. Most adverse events of amitriptyline are anticholinergic effects, such as constipation, dry mouth, drowsiness, cardiovascular effects, and orthostatic hypotension. Aripiprazole, which is a dopamine partial agonist, sometimes shows adverse events, such as sleep disorders, irritation, and tremor. Generally, BMS was found in the age of 50s−60s; however, the number of elderly patients with BMS are increasing recently (3). Very elderly patients who are over 80 years old are not uncommon nowadays. Chronic pain besides BMS is often found in elderly people. Many kinds of chronic pain, such as other chronic neuropathic pain, headache, chronic visceral pain, and chronic musculoskeletal pain, interact with each other and relate to anxiety and depression. Therefore, the pain symptoms in elderly patients tend to be complicated. Moreover, the risk of adverse events is high for elderly patients since the decline of hepatic activity and renal function leads to a higher medication concentration in plasma (4). The psychopharmacotherapy in elderly patients with BMS would be more complicated and difficult also because of multiple comorbid physical diseases, such as hypertension, cardiovascular diseases, diabetes, and osteoporosis (5). The selection of medication and decision of prescribing doses would be very difficult, although the dose of amitriptyline and dose of aripiprazole are usually very low in the treatment for BMS.

The aim of this study was to investigate the differences in the effectiveness and the adverse events of amitriptyline and aripiprazole focusing on very elderly patients primarily 80 years of age and over with BMS to facilitate the appropriate and effective treatment of BMS.

## Methods

### Participants

The patients with BMS who first visited our departments between April 1, 2017, and March 31, 2020, were involved in this study. The diagnoses of BMS were according to the criteria of the International Classification Headache Disorders-3 (1) and were performed by specialists in psychosomatic dentistry who were certified by the Japanese society of psychosomatic dentistry. Inclusion criteria were the patients who were (1) 80 years old or older, (2) started psychopharmacotherapy for BMS with amitriptyline or aripiprazole. The exclusion criteria were the patients (1) who had already undergone and kept getting the treatment with antidepressants from other clinics and (2) who visited our clinic only the day of the first examination. Before starting psychopharmacotherapy, routine consultation with their primary physicians about comorbid physical diseases was performed especially for patients who declared having glaucoma, prostatic hyperplasia, cardiovascular disease, and diabetes mellitus. After verifying that comorbid physical diseases had a low risk of adverse events by amitriptyline or aripiprazole, psychopharmacotherapy was started. For the selection of medications, comorbid physical diseases, somatic symptoms, functional impairment in cognitive function, work, and social activities were considered. When amitriptyline would be suspected not tolerable, aripiprazole was prescribed as a second-line medication. To prevent adverse events, the initial dose was set low and titrated slowly through every 1–2 weeks follow-up in the first month. From the second month to the end of the follow-up period, the presence of adverse events and the effectiveness were assessed with every 2–4 weeks follow-up. The collaboration between physicians and dentists remained throughout the follow-up period.

All clinical data, including sex, age, the duration of illness, the initial pain intensity (the initial visual analog scale; VAS), the salivation (the results of Saxon test), comorbid physical diseases, psychological questionnaires, the prescribed doses of amitriptyline or aripiprazole (initial, maximum and effective dose), clinical global improvement (CGI), and adverse events, were obtained from their medical charts.

### Assessments

The elderly patients often have a comorbidity of somatic symptoms, i.e., other chronic pain, which may make BMS symptoms more complicated. To assess the comorbid somatic symptoms, a somatic symptom scale-8 (SSS-8) was used (6). The scores from 0 (not at all) to 4 (very much) were marked for every 8 items as follows: stomach or bowel problems, back pain, pain in arms, legs or joints, headache, chest pain or shortness of breath, dizziness, feeling tired or having low energy, and trouble sleeping. The total scores and individual scores for each item were collected. For psychological questionnaires, the short intolerance of uncertainty scale (SIUS) was used to assess intolerance of uncertainty; Zung's self-rating depression scale (SDS) was used to assess depressive state; and pain catastrophizing scale (PCS) was used to assess pain catastrophizing. CGI was used to assess symptom severity (illness severity; CGI-1), improvement of BMS (global improvement; CGI-2), and adverse events by amitriptyline or aripiprazole (effectiveness index; CGI-3) (7). The prescription doses, of when BMS symptoms were assessed as “much improved” with CGI-2, were regarded as the effective dose. In addition, the patients assessed as “much improved” and “very much improved” were regarded as clinically improved. All examinations in this study were performed by well-experienced and trained clinicians and researchers. All data are shown average ± SD or median (interquartile range; IQR). For the analysis of the group differences, the Chi-square test, Student's *t*-test, Mann-Whitney U-test, and Kaplan-Meier (generalized Wilcoxon test) were performed by using IBM SPSS Statistics version 25.0 (IBM corporation, Armonk, NY, USA). Statistical significance was set at *p* < 0.05.

### Ethical Statements

All participants provided written informed consent. This study was approved by the Ethical Committee of Tokyo Medical and Dental University Dental Hospital (approval number: D2013-005).

## Results

**Figure 1:**
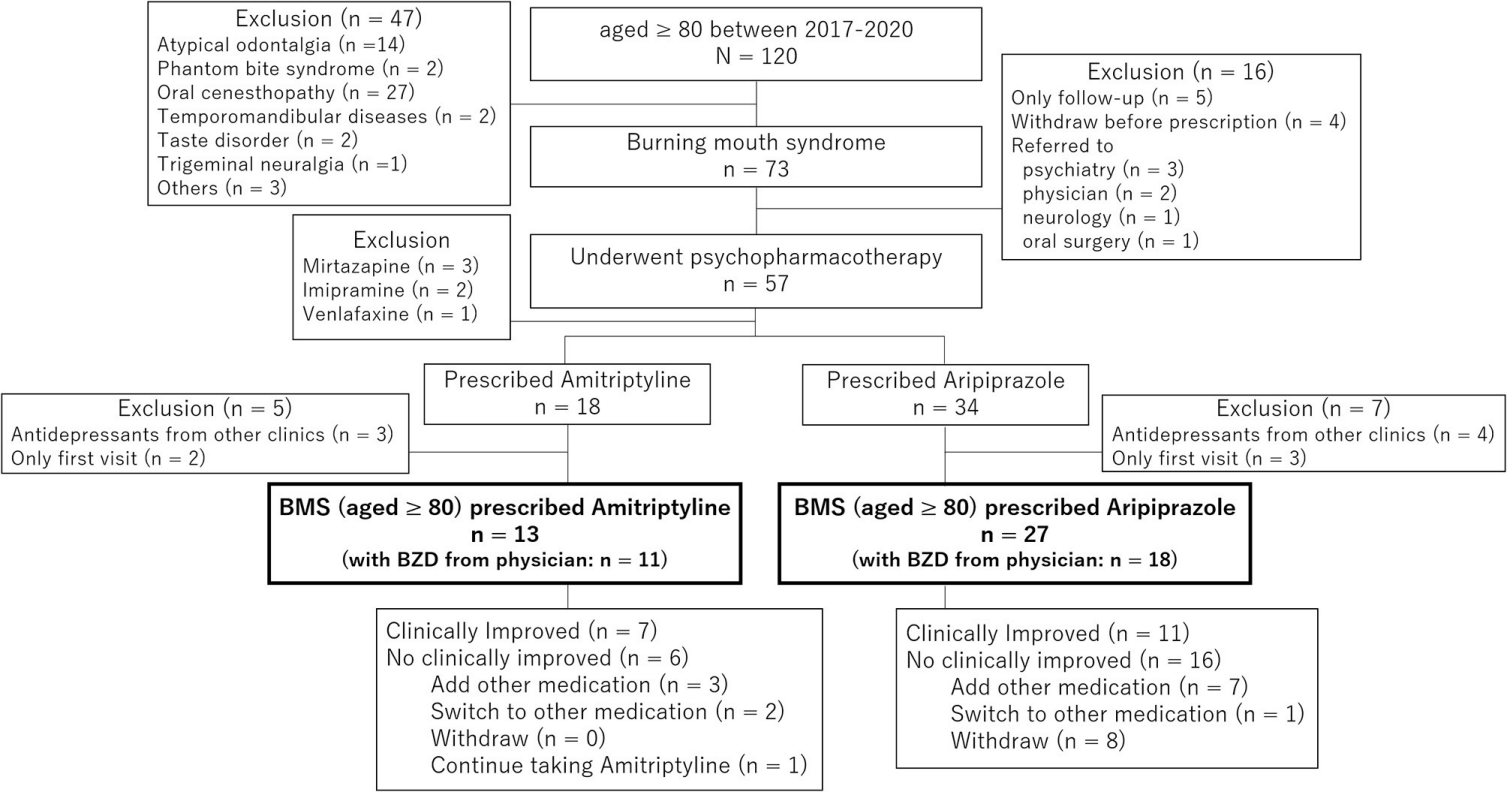
The chart of patients involved in the present study. In 73 patients with burning mouth syndrome (BMS) whose age was 80 or over, 57 patients underwent psychopharmacotherapy that include 18 patients who were prescribed amitriptyline and 34 patients who were prescribed aripiprazole. After exclusion of the duplicated prescriptions from other clinics and withdrawal, 13 patients who were prescribed amitriptyline and 27 patients who were prescribed aripiprazole were involved in the present study.

Of 1,568 new patients to our clinic between April 2017 and March 2020, 120 patients (7.7%) were 80 years old and above diagnosed with BMS (*n* = 73), atypical odontalgia (*n* = 14), phantom bite syndrome (*n* = 2), oral cenesthopathy (*n* = 27), and so on. In 73 patients with BMS, 57 patients underwent psychopharmacotherapy, including 18 patients who were prescribed amitriptyline and 34 patients who were prescribed aripiprazole. After exclusion of the duplicated prescriptions from other clinic and withdrawal, 13 patients (11 women, mean age; 82.3 ± 2.1 years old) who were prescribed amitriptyline and 27 patients (26 women, mean age; 84.2 ± 3.8 years old) who were prescribed aripiprazole were included in this study (Figure 1). As shown in Table 1, there is no significant between-group difference in sex (*p* = 0.242), age (*p* = 0.052), the duration of illness (46.2 ± 52.6 days vs. 58.0 ± 56.3 days, *p* = 0.520), the initial VAS (58.3 ± 20.0 vs. 69.3 ± 22.6, *p* = 0.133), and salivation (2.11 ± 0.71 g/2 min vs. 1.89 ± 0.99 g/2 min, *p* = 0.446). Almost all patients had comorbid physical diseases (13/13 vs. 26/27, *p* = 1.000). While the patients prescribed amitriptyline mostly had comorbidity of orthopedic diseases (*n* = 6, 46.2%) followed by hypertension (*n* = 5, 38.5%), cataract (*n* = 5, 38.5%), and gynecological diseases (*n* = 5, 38.5%), the patients prescribed aripiprazole had orthopedic diseases (*n* = 11, 40.7%), digestive diseases (*n* = 10, 37.0%), hypertension (*n* = 8, 29.6%), and cataract (*n* = 5, 18.5%). Moreover, there was no psychiatric comorbidity in 9 patients (69.2%) with amitriptyline and 16 patients (59.3%) with aripiprazole without a significant between-group difference (*p* = 0.730). Benzodiazepines had been prescribed to 11 patients (84.6%) with amitriptyline and 18 patients (66.7%) with aripiprazole by their primary physicians at the first examination in our clinic.

**Table 1:** The clinical differences between amitriptyline and aripiprazole in very elderly patients with BMS.

	**Amitriptyline (*n* = 13)**	**Aripiprazole (*n* = 27)**	***p*-values**
Sex [female (%)]	11 (84.6)	26 (96.3)	0.242
Age (years old)	82.3 ± 2.1	84.2 ± 3.8	0.052
Duration of illness (months)	46.2 ± 52.6	58.0 ± 56.3	0.520
Initial VAS	58.3 ± 20.0	69.3 ±22.6	0.133
Salivation, Saxon test (g/2 min)	2.11 ± 0.71	1.89 ± 0.99	0.446
**Comorbid physical diseases [*****n*** **(%)]**			
Absent	0 (0.0)	1 (3.7)	1.000
Present	13 (100.0)	26 (96.3)	
Hypertension	5 (38.5)	8 (29.6)	
Hyperlipidemia	2 (15.4)	4 (14.8)	
Diabetes mellitus	0 (0.0)	2 (7.4)	
Cataract	5 (38.5)	5 (18.5)	
Glaucoma	2 (15.4)	2 (7.4)	
Angina pectoris	0 (0.0)	1 (3.7)	
Other cardiovascular disease	0 (0.0)	4 (14.8)	
Orthopedic diseases	6 (46.2)	11 (40.7)	
Gynecological diseases	5 (38.5)	3 (11.1)	
Benign prostatic hyperplasia	1 (7.7)	0 (0.0)	
Digestive disease	2 (15.4)	10 (37.0)	
Insomnia	3 (23.1)	0 (0.0)	
Cerebrovascular disease	2 (15.4)	1 (0.3)	
Others	8 (61.5)	14 (51.9)	
**Comorbid psychiatric disorders [*****n*** **(%)]**			
Absent	9 (69.2)	16 (59.3)	0.730
Present	4 (30.8)	11 (40.7)	
Depression	1 (7.7)	1 (3.7)	
Anxiety disorder	0 (0.0)	3 (11.1)	
Insomnia	1 (7.7)	3 (11.1)	
Unknown details	2 (15.4)	4 (14.8)	
**Psychosomatic examinations (average** **±SD)**			
SIUS	30.5 ± 5.7	27.7 ± 9.7	0.349
SDS	43.1 ± 8.8	44.3 ± 11.1	0.718
PCS	29.9 ± 10.4	37.7 ± 10.5	**0.042**
STarTG	1.7 ± 1.5	2.7 ± 1.3	0.050
SSS-8	8.1 ± 5.6	11.2 ± 4.8	0.097
**Prognosis of psychopharmacotherapy [median (IQR)]**			
Initial dose (mg)	5.0 (5.0, 5.0)	0.3 (0.3, 0.3)	
Maximum dose (mg)	15.0 (5.0, 20.0)	0.75 (0.5, 1.0)	
Duration of taking medication (months)	491 (275, 567)	89 (40.5, 246)	**0.021**
CGI-1: illness severity	4 (4,4)	4 (4,4)	0.289
CGI-2: global improvement	2 (2,3)	3 (2,4)	0.407
CGI-3: effectiveness index	6 (6,10)	9 (5,13)	0.864
Improved by monotherapy	7 (53.8)	11 (40.7)	0.329
Effective dose (mg, *n* = 7 vs. *n* = 11)	10 (7.5, 15.0)	1.0 (0.5, 1.5)	
Duration until clinical improvement (months, *n* = 7 vs. *n* = 11)	105 (40.5, 181.5)	80 (29.5, 102)	0.395
**Adverse events [*****n*** **(%)]**			
Absent	4 (30.8)	20 (74.1)	**0.015**
Present	9 (69.2)	7 (25.9)	
Constipation	6 (46.2)	0 (0.0)	
Dizziness	5 (38.5)	1 (3.7)	
Dry mouth	4 (30.8)	0 (0.0)	
Drowsiness	3 (23.1)	1 (3.7)	
Dysuria	2 (15.4)	0 (0.0)	
Sleep disorders	0 (0.0)	3 (11.1)	
Loss of apatite	0 (0.0)	2 (7.4)	
Irritability	0 (0.0)	1 (3.7)	
Headache	0 (0.0)	1 (3.7)	

*SD, standard deviation; IQR, interquartile range; VAS, visual analog scale; SIUS, short intolerance of uncertainty scale; SDS, Zung's self-rating depression scale; PCS, pain catastrophizing scale; STarT-G, subgrouping for targeted treatment generic; SSS-8, somatic symptom scale 8; CGI, clinical global impression; bold p values, p < 0.05*.

The scores of pain catastrophizing scale (PCS) were significantly higher in the patients of aripiprazole compared to the one in the patients of amitriptyline (*p* = 0.042), while no between-group significance was observed in the short intolerance of uncertainty scale (SIUS; *p* = 0.349), Zung's self-rating depression scale (SDS; *p* = 0.718), and subgrouping for targeted treatment generic (STarT-G; *p* = 0.050).

**Figure 2:**
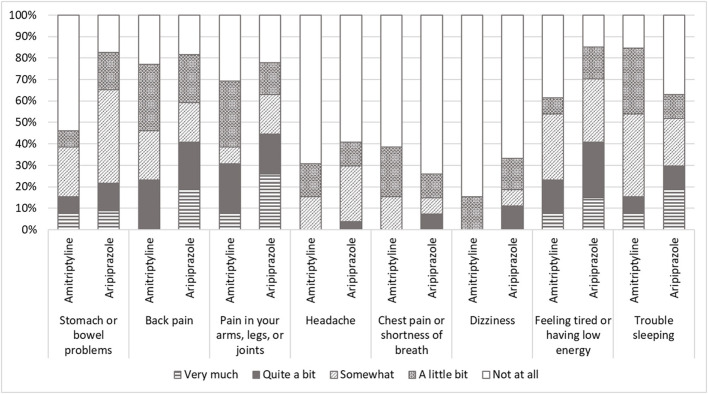
The scores of somatic symptom scale-8 (SSS-8). While most comorbid somatic symptom was “trouble sleeping” in the patients with amitriptyline, “stomach or bowel problems” and “feeling tired or having low energy” were the most in the patients with aripiprazole. “Back pain” and “pain in arms, legs, or joints” were also observed in many patients in both patients with amitriptyline and with aripiprazole.

Furthermore, the different tendency between the groups was observed in the parameters of SSS-8 although no significant difference of total scores was observed (*p* = 0.097). While the most common comorbid somatic symptom was “trouble sleeping” in the patients treated with amitriptyline, “stomach or bowel problems” and “feeling tired or having low energy” were the most common in the patients treated with aripiprazole (Figure 2). “Back pain” and “pain in arms, legs, or joints” were also observed in many patients in both groups.

**Figure 3:**
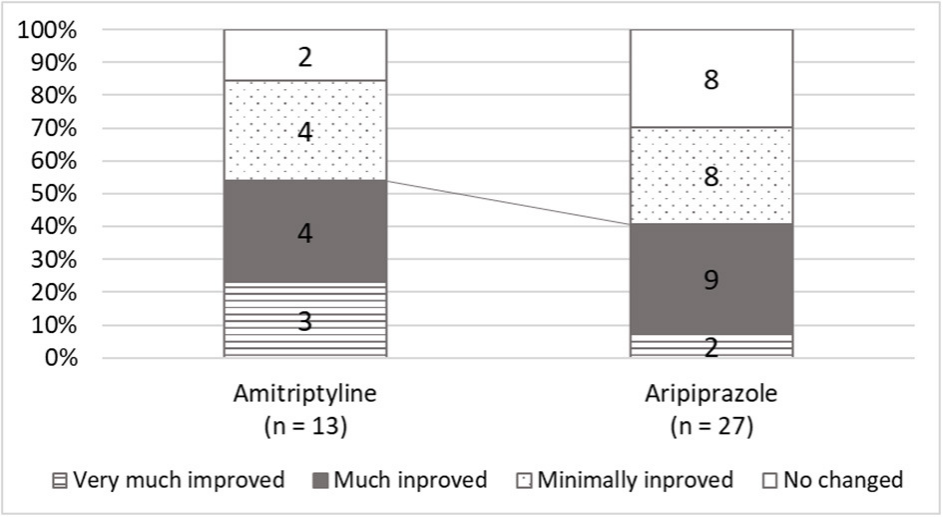
The scores of global improvements in clinical global improvement (CGI). While amitriptyline was responded to in 84.6% of patients and clinically improved 53.8% of patients, aripiprazole was effective in 70.4%, and 40.7% of patients showed clinical improvement.

The initial doses of amitriptyline were 2.5 or 5 mg [5.0 (5.0, 5.0) mg] and the median of the maximum doses was 15 (5.0, 20.0) mg. The effective dose of amitriptyline 10 (7.5, 15.0) and the median duration of taking amitriptyline until “much improvement” was 105 (40.5, 181.5) days. For aripiprazole, the initial prescribed dose was 0.3 (0.3, 0.3) mg and titrated up to a maximum dose of 0.75 (0.5, 1.0) mg. The effective dose of aripiprazole was 1.0 (0.5, 1.5) mg, and the duration until clinical improvement was 80 (29.5, 102) days. While amitriptyline represented clinically improvement in 7 patients (53.9%), aripiprazole represented in 11 patients (40.4%) without a significant between-group difference (Figure 3). In 16 patients who did not clinically improve with aripiprazole, 7 patients were added to other medication, 1 patient was switched to other medication, and 8 patients withdrew the treatment.

The adverse events of amitriptyline were observed in 9 patients (69.2%) with the most complains of constipation (*n* = 6) followed by dizziness (*n* = 5), dry mouth (*n* = 4), drowsiness (*n* = 3), and dysuria (*n* = 2). On the other hand, 7 patients (25.9%) reported adverse events with aripiprazole, and most were sleep disorders (*n* = 3). Aripiprazole represented significantly fewer adverse events than amitriptyline (*p* = 0.015).

**Figure 4:**
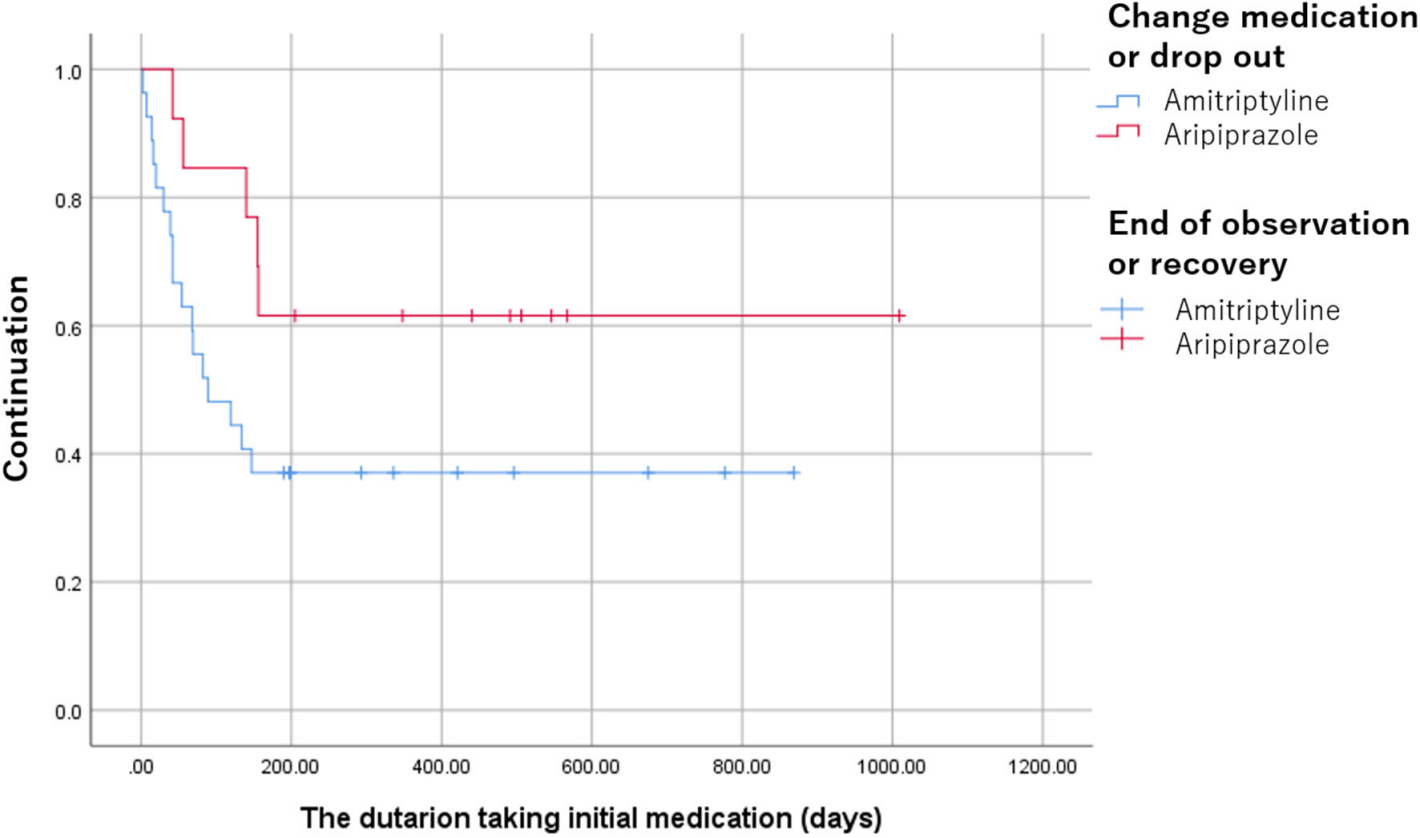
The discontinuation of amitriptyline and aripiprazole. The discontinuation was significantly lower in the patients with amitriptyline compared to the patients with aripiprazole (*p* = 0.043).

In the patients with amitriptyline, the duration of taking medication was observed significantly longer [491 (275, 567) days vs. 89 (40.5, 246) days, *p* = 0.021] and discontinuation was significantly lower compared to the patients with aripiprazole (*p* = 0.043, Figure 4).

## Discussion

This is the first comparative study that investigated the effectiveness and adverse events of amitriptyline and aripiprazole for patients with BMS, focusing on very elderly patients. While amitriptyline clinically improved 53.8% (7/13) patients, aripiprazole was effective in 40.7% (11/27) patients with low initial doses and slow titration. Amitriptyline showed significantly more adverse events (69.2%) than aripiprazole (25.9%); however, no withdrawal due to adverse events were observed in both groups. Moreover, the discontinuation of amitriptyline was significantly lower than aripiprazole.

Amitriptyline is the first-line medication of psychopharmacotherapy for BMS (2) which is the same as treatment for other chronic pain (8). Besides, aripiprazole was also reported as another effective medication for BMS. However, the adverse events associated with these medications are sometimes troublesome, especially in elderly patients. According to the aging society in Japan, the patients with BMS are aging recently (3, 5). In elderly patients, the pharmacokinetics, including drug-metabolism and elimination, were decreased, and higher plasma medication levels would be induced (4). Moreover, comorbid physical diseases, including various somatic symptoms that elderly patients generally have, may exacerbate and complicate not only BMS symptoms but also adverse events. Therefore, the risk related to the various adverse events would be higher in the elderly patients (5) and make a selection of medications difficult. In the present study, adverse events were observed more in the patients with amitriptyline (69.2%) than in the patients with aripiprazole (25.9%) as we had hypothesized. However, there was no patient who withdrew because of severe adverse events. This might be a result of the low initial dose use and slow titration in the treatment for BMS.

In the present study, anticholinergic effects, such as constipation, dizziness, dry mouth, and drowsiness, were observed in the patients treated with amitriptyline while sleep disorders were observed in the patients treated with aripiprazole. Constipation is mostly found in patients with amitriptyline and sometimes becomes troublesome because it is often already seen in elderly people. Since no patient with aripiprazole complained of constipation, aripiprazole would be another choice for patients who have comorbid digestive system disorders. Bristol stool chart (9) might be useful to understand the statement of constipation at the decision of medication and during careful follow-up. Dry mouth is also one of the major adverse events of amitriptyline. In the present study, although there was no significant difference, the patients with aripiprazole represent less salivation than the patients with amitriptyline and the reference value (2.0 g/2 min) at the initial examination. It may reflect the selection bias that the prescription of amitriptyline might be avoided for patients with hyposalivation. On the other hand, subjective dry mouth is one of the specific symptoms besides the burning sensation in BMS. Moreover, taste disturbance is also found as not only one of the adverse events but also a partial symptom of BMS (2). It may be found as an aging physiological change in some elderly people. Therefore, regular quantitative examinations, such as Saxon's test and gustatory examination, are needed to assess objective dry mouth and taste disturbance. For dizziness and orthostatic hypotension, a higher risk of fall was observed in the elderly patients who were taking antipsychotics, antidepressants, or benzodiazepines in the previous reports (10). In the present study, there were no patients who fall or got injured; however, the explanation about the risk of fall to patients' families besides the patient herself/himself is important to prevent accidents. Neither delirium nor hallucination was observed in this study; however, these symptoms also should be considered as adverse events. In very elderly patients, it is difficult to distinguish whether the symptoms are caused by side effects or by other diseases, such as dementia. Moreover, a case with the oral psychosomatic symptoms in which Levy body dementia developed during treatment was also reported (11). Careful follow-up with MRIs may be needed not to mention the Hasegawa dementia scale-revised and mini-mental state examination.

At the same time, since amitriptyline induces drowsiness, taking medication at bedtime is generally recommended. For the cases drowsiness is shown daytime, taking in the early evening would be suggested. For amitriptyline, the complaining rate of drowsiness was lower in 80 years old and older patients (23.1%) in this study than those under 65 years old patients (57.5%), 65–75 years old patients (48.1%), and over 75 years old patients (36.4%) in our previous study (5). On the other hand, sleep disorders were observed in 3 patients (11.1%) with aripiprazole. In Japan, many elderly people were prescribed benzodiazepine for reasons of insomnia, anxiety, and so on (12). In the present study, 84.6% (11/13) of the patients with amitriptyline and 66.7% (18/27) had been taking benzodiazepines and 70.0% (28/40) of the patients complained of sleep disturbance in SSS-8. While special attention to sleep conditions would be required to prescribe amitriptyline and aripiprazole. The improvement of sleep disturbance due to taking amitriptyline would make drowsiness rather beneficial for these patients with sleep disturbance (13). In SSS-8, the patients with amitriptyline represented trouble sleeping mostly, less stomach and bowel problems, and feeling tired. Moreover, the effect of medication, various adverse events are also found different in individuals. Individualized treatment is critical especially for very elderly patients with BMS. Not only the medical history but also SSS-8 may be useful to understand comorbid patients' somatic symptoms in other body parts, which may relate to the complicated BMS symptoms and may be helpful to avoid adverse events caused by medication. Moreover, PCS might be another predictor for the selection of medications since the patients treated with aripiprazole showed significantly higher scores in PCS than the patients with amitriptyline. However, there was no significant difference in other psychological questionnaires. Further study with more samples is needed to investigate the relation between the difference of medications and psychological states.

While amitriptyline is prescribed in depressed patients with a dose of 150–300 mg, it is prescribed in patients with chronic pain with 10–25 mg initially and titrated to 150 mg (8). The effective dose for very elderly patients with BMS was 10 mg and started with a lower dose, 2.5 or 5.0 mg. Similarly, while aripiprazole is generally prescribed 12–24 mg for schizophrenia or bipolar disorders, the initial dose was 0.5 mg and the effective dose was 1.0 mg in the present study. A lower dose of amitriptyline and aripiprazole would be effective for psychopharmacotherapy of BMS, especially in elderly patients. Necessity minimum but sufficient effective doses of medications are also required to avoid adverse events.

Furthermore, amitriptyline has been reported its problematic adverse events with higher withdrawal than other antidepressants while aripiprazole has been reported as a useful antipsychotic with better tolerability. Interestingly, the present study revealed that amitriptyline has lower discontinuation than aripiprazole despite the higher occurrence rate of adverse events. The low dose amitriptyline monotherapy may be effective for BMS in safety with better tolerability over the collaboration with primary physician despite some adverse events. In addition, 59.3% of patients with aripiprazole quit or switched to other medication or augmentation with other antidepressants requiring more improvements in the early period of treatments similar to our previous report (14). Aripiprazole might have a limitation as monotherapy but might be more effective as augmentation with other antidepressants. If the well-balanced polypharmacotherapy, which has a synergistic effect with fewer adverse events, could be defined, it will be another choice for very elderly patients.

There are a few limitations to this study. First, the sample size is small without a placebo arm. Further investigation with a larger sample size considering a placebo arm is required to analyze the effectiveness and adverse events of antidepressants. Second, the quantitative examinations were lacking. During the follow-up duration, blood tests that include the concentration of medication in plasma level and salivation were not constantly performed by practitioners. However, all participants had been kept attending their physicians and regularly performed blood tests if necessary, and the collaboration between physicians and dentists had also kept throughout the treatment and the effects on comorbid physical diseases were not observed. Third, the bias at the decision for the first prescription of amitriptyline or aripiprazole might exist since this is a retrospective study.

In conclusion, for the very elderly BMS patients of 80 years and over, both treatments with the low initial doses and slow titration of amitriptyline and aripiprazole were effective. Moreover, amitriptyline showed significantly lower discontinuation despite a higher occurrence rate of adverse events than aripiprazole. Therefore, aripiprazole may have some advantages in the adverse events compared to amitriptyline; however, the low dose amitriptyline monotherapy may have more benefit in the effectiveness and tolerability over prudent collaboration with primary physicians.

## Data Availability Statement

The original contributions presented in the study are included in the article, further inquiries can be directed to the corresponding author.

## Ethics Statement

The studies involving human participants were reviewed and approved by the Ethical Committee of Tokyo Medical and Dental University Dental Hospital. The patients provided their written informed consent to participate in this study.

## Author Contributions

MW participated in data acquisition, analysis, and writing the manuscript. CT, ZL, GN, TS, CH, TT, TY, and MT participated in collecting data. HM, TN, and AT developed the theory and supervised the research. AT designed the research and administrated project. All authors read and approved the final manuscripts.

## Funding

This research was funded by KAKENHI from the Japanese Society for the Promotion of Science (JSPS), grant number 19K10328 to AT and grant number 19K19211 to MW. The funder had no role in study design, data collecting, data analysis, the decision of publishing, and preparation of the manuscript.

## Conflict of Interest

The authors declare that the research was conducted in the absence of any commercial or financial relationships that could be construed as a potential conflict of interest.

## Publisher's Note

All claims expressed in this article are solely those of the authors and do not necessarily represent those of their affiliated organizations, or those of the publisher, the editors and the reviewers. Any product that may be evaluated in this article, or claim that may be made by its manufacturer, is not guaranteed or endorsed by the publisher.
